# Characterization of the gut microbiota in people with different levels of obesity

**DOI:** 10.3389/fmicb.2025.1679119

**Published:** 2025-12-03

**Authors:** Ergan Li, Senlin Wang, Youqin Li, Anke Liuli, Meifang Liang, Jian Huang, Yan Li, Haifang Li, Zhonghui Feng

**Affiliations:** 1 The Third People's Hospital of Chengdu, Chengdu, Sichuan, China; 2 College of Animal Science and Veterinary Medicine, Southwest Minzu University, Chengdu, China; 3 College of Medicine, Southwest Jiaotong University, Chengdu, China; 4 College of Life Science and Engineering, Southwest Jiaotong University, Chengdu, China; 5 Key Laboratory of Animal Medicine, Southwest Minzu University, Chengdu, Sichuan, China; 6 Veterinary Teaching Hospital, Southwest Minzu University, Chengdu, China; 7 Department of Clinical Veterinary Medicine, College of Animal Science and Veterinary Medicine, Southwest Minzu University, Chengdu, Sichuan, China; 8 College of Life Science, Shandong Agricultural University, Tai'an, China; 9 The Center of Obesity and Metabolic Diseases, Department of General Surgery, The Third People's Hospital of Chengdu, The Affiliated Hospital of Southwest Jiaotong University, Chengdu, Sichuan, China

**Keywords:** degree of obesity, fecal microbiota, 16S rRNA sequencing, metagenomics, body mass index

## Abstract

**Purpose:**

With the evolution of dietary habits, obesity has emerged as a significant global health issue. Numerous studies have demonstrated a close association between obesity and gut microbiota; however, the specific contribution of gut microbiota to varying degrees of obesity remains inadequately understood. Consequently, this study aims to characterize the gut microbiota of individuals across different obesity severity levels.

**Methods:**

We conducted a comprehensive characterization of the gut microbiome in Chinese obese patients and a healthy control group through the application of 16S rRNA gene sequencing, supplemented by metagenomic sequencing. The study cohort was stratified into five distinct categories based on body mass index (BMI): healthy, overweight, and obesity grades I, II, and III.

**Results:**

In obese populations, the gut microbiome structure shifted significantly, with beneficial genera like *Faecalibacterium, Roseburia*, and *Ruminococcus* decreasing, and potentially harmful genera such as *Blautia, Collinsella*, and *Streptococcus* increasing. These changes impacted host metabolic pathways, including ribosome synthesis, RNA polymerase activity, and DNA repair. Clinical analyses also revealed strong links between specific genera and metabolic markers like lipid metabolism and insulin resistance.

**Conclusion:**

Populations with different obesity traits show unique changes in gut flora. The level of dysbiosis, or imbalance in intestinal microbiota, rises with obesity. These microbial changes are linked to host metabolism, indicating that targeting harmful bacteria and supplementing with beneficial ones from normal-weight populations could effectively reduce obesity.

## 1 Introduction

The transformation in dietary patterns, particularly the increasing prevalence of high-calorie, high-fat, and high-sugar diets, has contributed to the escalating issue of obesity, which has reached epidemic levels within the past decade and poses a significant challenge to global public health (Isolauri and Laitinen, 2025). According to the World Health Organization (WHO), over 1.9 billion adults worldwide are classified as overweight, with 650 million of these individuals meeting the criteria for obesity (World Health Organization, 2000; Liu et al., 2025). Obesity not only adversely affects individual quality of life but is also strongly linked to a range of chronic diseases, including type 2 diabetes (T2D), cardiovascular disease (CVD), hypertension (HTN), neurological disorders, chronic respiratory disorders, digestive disorders, and various cancers (Maxim et al., 2025; Mayer et al., 2021). These conditions are frequently associated with dysregulation of the gut microbial community.

In China, the body mass index (BMI) is extensively utilized for the classification and evaluation of obesity (Gulati and Murphy, 2025). Nonetheless, various criteria and research findings have indicated that the applicability and accuracy of BMI across different populations remain contentious. A particular study highlighted discrepancies between the BMI criteria employed for assessing overweight and obesity in Chinese populations and those established by the World Health Organization (WHO). Specifically, the WHO defines overweight as a BMI ≥ 25 kg/m^2^ and obesity as a BMI ≥ 30 kg/m^2^, whereas the Chinese working group's criteria categorize overweight as a BMI ≥ 24 kg/m^2^ and obesity as a BMI ≥ 28 kg/m^2^. These divergent criteria significantly influence the prediction of non-communicable diseases (NCDs) and multimorbidity, with the application of the more stringent WHO criteria potentially offering enhanced benefits in terms of cardiovascular metabolism (Fan et al., 2025). Consequently, obesity is classified into grades I, II, and III according to the WHO's BMI standards (World Health Organization, 2000). Grade I obesity represents the initial phase of obesity, while Grade II obesity (moderate obesity) can exacerbate or precipitate obesity-related comorbidities, including diabetes mellitus, hypertension, and other chronic conditions. Grade III obesity (severe obesity) is associated with the onset of multiple chronic diseases and an elevated risk of mortality. A comprehensive understanding of these obesity classifications and gradations enables individuals to evaluate their own obesity status and implement timely interventions to prevent the progression of obesity and its detrimental impact on health and quality of life.

In recent years, a growing body of research has demonstrated the significant role of the gut microbiota in both the development and reversal of obesity (De Wit et al., 2023). The gut microbiota contributes to the onset and progression of obesity through mechanisms that affect inflammatory responses, appetite regulation, and metabolic processes (Ma and Lee, 2025; Deng et al., 2025; Mishra et al., 2023). Notably, the gut microbiota in obese individuals is characterized by reduced diversity and an imbalance in the proportions of specific microbial communities. Furthermore, the gut microbiota can modulate the host's energy balance and fat storage by producing metabolites such as short-chain fatty acids (SCFAs) (Aja et al., 2025). As a complex ecosystem within the human body, the gut microbiota participates in a variety of physiological processes, including nutrient absorption, metabolite synthesis, immune system regulation, and energy metabolism, thereby exerting a profound influence on the development of various diseases (including obesity) (Doghish et al., 2025; Zhang and Wang, 2024). Consequently, it is imperative to investigate the characteristics of the gut microbiota as a marker for the progression of obesity across individuals with varying degrees of obesity.

While numerous studies have investigated the relationship between obesity and the gut microbiota, there is a paucity of research focusing on the characteristics of the gut microbiota across varying degrees of obesity. Consequently, this study aims to compare the composition and diversity of the gut microbiota among individuals with different obesity levels using 16S rRNA gene sequencing and metagenomic sequencing. The objective is to systematically characterize the gut microbiota of Chinese patients with varying obesity levels, alongside healthy control cohorts, and to explore the potential mechanisms by which the gut microbiota may influence the progression of obesity. This research seeks to provide a novel scientific basis for obesity prevention and treatment, thereby laying the groundwork for the development of personalized medical strategies.

## 2 Materials and methods

### 2.1 Research participants and design

In accordance with the World Health Organization (WHO) criteria, the 36 study participants were categorized into five distinct groups based on their Body Mass Index (BMI): normal weight (*N* = 8, 18.5 ≤ BMI < 24.9 kg/m^2^), overweight (*N* = 7, 25.0 ≤ BMI < 29.9 kg/m^2^), grade 1 obesity (*N* = 8, 30.0 ≤ BMI < 34.9 kg/m^2^), grade 2 obesity (*N* = 8, 35.0 ≤ BMI < 39.9 kg/m^2^), and grade 3 obesity (*N* = 5, BMI ≥ 40.0 kg/m^2^). The obese participants were selected from patients scheduled for bariatric surgery between September 2019 and October 2020 at the Third People's Hospital of Chengdu City, while the control group consisted of healthy volunteers. A majority of the obese patients presented with comorbid conditions such as hypertension, hyperglycemia, fatty liver, type 2 diabetes mellitus, or obstructive sleep apnea syndrome. Furthermore, the study collected clinical indicators, including alanine aminotransferase (ALT), aspartate aminotransferase (AST), high-density lipoprotein (HDL), low-density lipoprotein (LDL), total cholesterol (TC), fasting C-peptide (FCP), insulin, fasting blood glucose (FBG), and triglycerides (TG) from the hospital database, to examine the relationship between obesity and gut microbiota.

### 2.2 Fecal DNA extraction, PCR amplification, and 16S rRNA sequencing

Following storage of the fecal samples at −80°C, microbial genomic DNA was extracted utilizing the FastPure Stool DNA Isolation Kit (MJYH) and subsequently assessed for quality via 1% agarose gel electrophoresis. The V3-V4 region of the 16S rRNA gene was amplified using the primers 338F/806R. Post library construction with the NEXTFLEX Rapid DNA-Seq Kit, sequencing was conducted on the Illumina NextSeq 2000 PE300 platform (MJYH).

### 2.3 Statistical analysis

**Figure 1:**
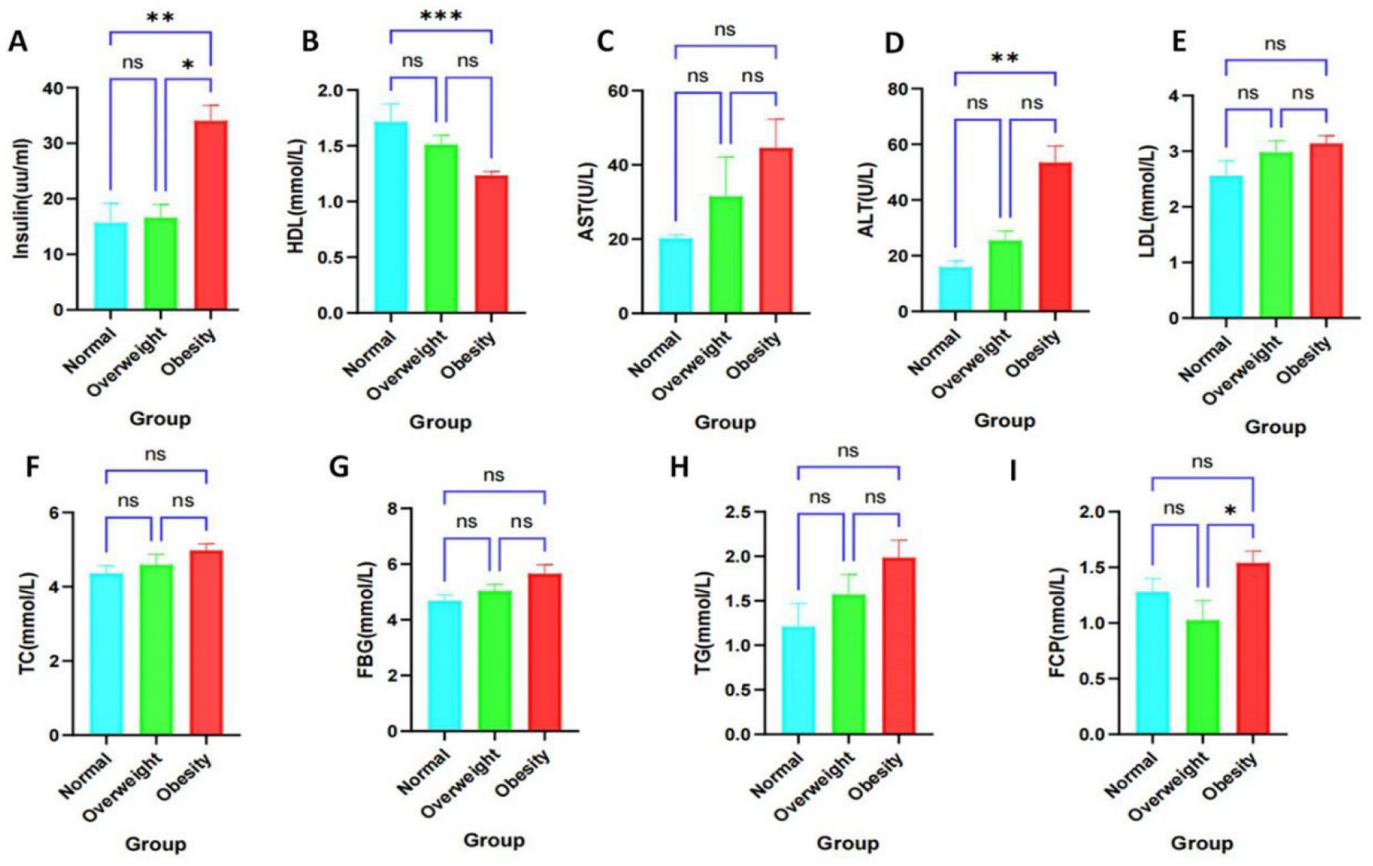
Clinical factor analysis of healthy, overweight and obese populations. *P < 0.05, **P < 0.01, ***P < 0.001. ns, not significantly different. **(A)** High-density lipoprotein. **(B)** Fasting insulin levels. **(C)** Aspartate aminotransferase. **(D)** Alanine aminotransferase. **(E)** Low-density lipoprotein. **(F)** Total cholesterol. **(G)** Fasting glucose. **(H)** Triglycerides. **(I)** Fasting C-peptide.

All data analyses were conducted using the Meggie BioCloud platform (https://cloud.majorbio.com). For alpha diversity, indices such as Chao1 and Shannon were calculated utilizing mothur (version 1.30.2), and inter-group differences were assessed using the Wilcoxon rank-sum test. Regarding beta diversity, Principal Coordinates Analysis (PCoA) was performed based on the Bray-Curtis distance, and differences in microbial community structure between groups were evaluated using PERMANOVA. Species Venn diagram analyzed using Python 2.7.10 software. For differential species analysis, the LEfSe method (with LDA > 4 and *P* < 0.05) was employed to identify significant differences in flora from the phylum to genus levels. Figure 1 clinical factor differences were analyzed using Prism version 10. The table of baseline patient characteristics by obesity category (Table 1) was generated using R software.

**Table 1:** Baseline patient characteristics by obesity category.

**Characteristics**	**Overall**	**Control**	**Overweight**	**ClassI**	**ClassII**	**ClassIII**	***p***
*n*	36	8	7	8	8	5	
Gender = female (%)	23 (63.89)	6 (75.00)	4 (57.14)	5 (62.50)	5 (62.50)	3 (60.00)	0.9617
Age [mean (SD)]	36.36 (9.85)	39.12 (10.20)	41.57 (10.97)	30.62 (8.05)	36.62 (10.34)	33.40 (6.80)	0.2749
BMI [mean (SD)]	31.81 (7.59)	21.89 (2.39)	27.79 (1.03)	31.92 (1.44)	37.62 (1.11)	43.82 (3.60)	< 0.0001
ALT [mean (SD)]	57.08 (79.01)	13.88 (5.57)	89.57 (135.44)	48.01 (21.79)	90.04 (99.65)	42.50 (25.78)	0.002
AST [mean (SD)]	36.80 (48.94)	20.38 (3.07)	53.29 (82.24)	28.14 (10.47)	55.99 (69.32)	23.14 (7.74)	0.2996
HDL [mean (SD)]	1.29 (0.29)	1.57 (0.34)	1.33 (0.26)	1.21 (0.18)	1.15 (0.22)	1.16 (0.20)	0.0239
LDL [mean (SD)]	3.07 (1.01)	3.13 (1.17)	3.07 (1.07)	3.04 (1.22)	2.86 (0.84)	3.34 (0.90)	0.8812
TC [mean (SD)]	4.85 (1.09)	4.53 (0.61)	5.12 (1.11)	5.01 (1.61)	4.58 (0.88)	5.17 (1.17)	0.6573
FCP [mean (SD)]	1.38 (0.48)	1.28 (0.34)	1.03 (0.46)	1.56 (0.62)	1.57 (0.45)	1.47 (0.31)	0.2473
Insulin [mean (SD)]	27.01 (14.60)	18.11 (10.77)	19.81 (5.24)	34.89 (18.08)	33.90 (17.91)	27.68 (4.42)	0.0282
FBG [mean (SD)]	5.48 (2.27)	4.88 (0.76)	5.07 (0.54)	4.80 (0.50)	7.23 (4.43)	5.35 (0.85)	0.1432
TG [mean (SD)]	1.93 (1.37)	1.22 (0.55)	2.17 (1.24)	2.02 (1.13)	2.41 (2.32)	1.82 (0.54)	0.094

SD, standard deviation; ALT, alanine aminotransferase; AST, aspartate transaminase; HDL, high-density lipoprotein; LDL, low-density lipoprotein; BMI, body mass index; TC, total cholesterol; FCP, fasting plasma c-peptide; FBG, fasting blood glucose; TG, triglyceride.

### 2.4 DNA extraction, library construction, and macro-genomics sequencing of fecal samples

Prior to the extraction and analysis of deoxyribonucleic acid (DNA), all fecal samples were frozen at −80 C. The methods employed for DNA extraction and the quality control procedures were identical to those utilized for 16S rRNA sequencing. The DNA was fragmented using the Covaris M220 (Genetics, China), and following screening of fragments measuring approximately 350 base pairs, libraries were constructed using the NEXTFLEX Rapid DNA-Seq (Bioo Scientific, USA). Subsequent to this, sequencing was performed using the Illumina NovaSeq™ X Plus (Illumina, USA) sequencing platform. The macrogenome sequencing procedure was carried out by Shanghai Meiji Biomedical Technology Co., Ltd.

### 2.5 Macrogenomics sequence quality control, genome assembly, and gene prediction

Before the extraction and analysis of DNA, all fecal samples were preserved by freezing at −80 C. The methods for DNA extraction and quality control adhered to established protocols. Quality control of DNA sequences was conducted using fastp (version 0.20.0), and sequence alignment was performed with BWA (version 0.7.17). MEGAHIT (version 1.1.2) facilitated sequence assembly, with contigs of ≥300 base pairs being selected as the final output. Open reading frames (ORFs) were predicted using Prodigal (version 2.6.3), with genes of ≥100 base pairs being retained and subsequently translated into amino acid sequences. To construct non-redundant gene sets, CD-HIT (version 4.7) was employed, while SOAPaligner (version 2.21) was utilized to quantify gene abundance.

### 2.6 Species and KEGG functional annotation

Sequence alignment analysis was conducted utilizing Diamond version 2.0.13. Amino acid sequences from non-redundant gene sets were aligned to the NR database using BLASTP with an e-value threshold of 1e-5, facilitating the calculation of species abundance in conjunction with taxonomic annotations. Similarly, these sequences were aligned to the KEGG database using BLASTP with the same e-value threshold, enabling the determination of functional class abundance based on KO, Pathway, EC, and Module annotations.

## 3 Results

### 3.1 Baseline characterization of patients in different obesity categories

We conducted an analysis of the biochemical profiles of obese individuals using collected clinical data to enhance diagnostic accuracy and clinical support. Specifically, we examined notable differences in levels of AST, ALT, HDL, LDL, TC, FCP, Insulin, FBG, and TG. Our findings indicated that HDL levels were significantly lower (Figure 1A), while Insulin levels were significantly higher (Figure 1B) in obese individuals compared to healthy controls. No significant differences were observed in AST, ALT, LDL, TC, FCP, FBG, and TG levels; however, there was a trend toward increased levels of AST, ALT, TC, FBG, and TG (Figures 1C–H). Additionally, FCP levels were significantly elevated in obese subjects compared to overweight subjects, but not significantly different from healthy individuals (Figure 1I). The baseline characteristics of patients, categorized by obesity status (Table 1), revealed significant differences in BMI, ALT, HDL, and Insulin among obese subgroups (*P* < 0.05). These findings suggest that obese patients are at an increased risk for insulin resistance, metabolic disorders, and obesity-related complications.

### 3.2 Structural changes in the gut microbiota of obese patients

**Figure 2:**
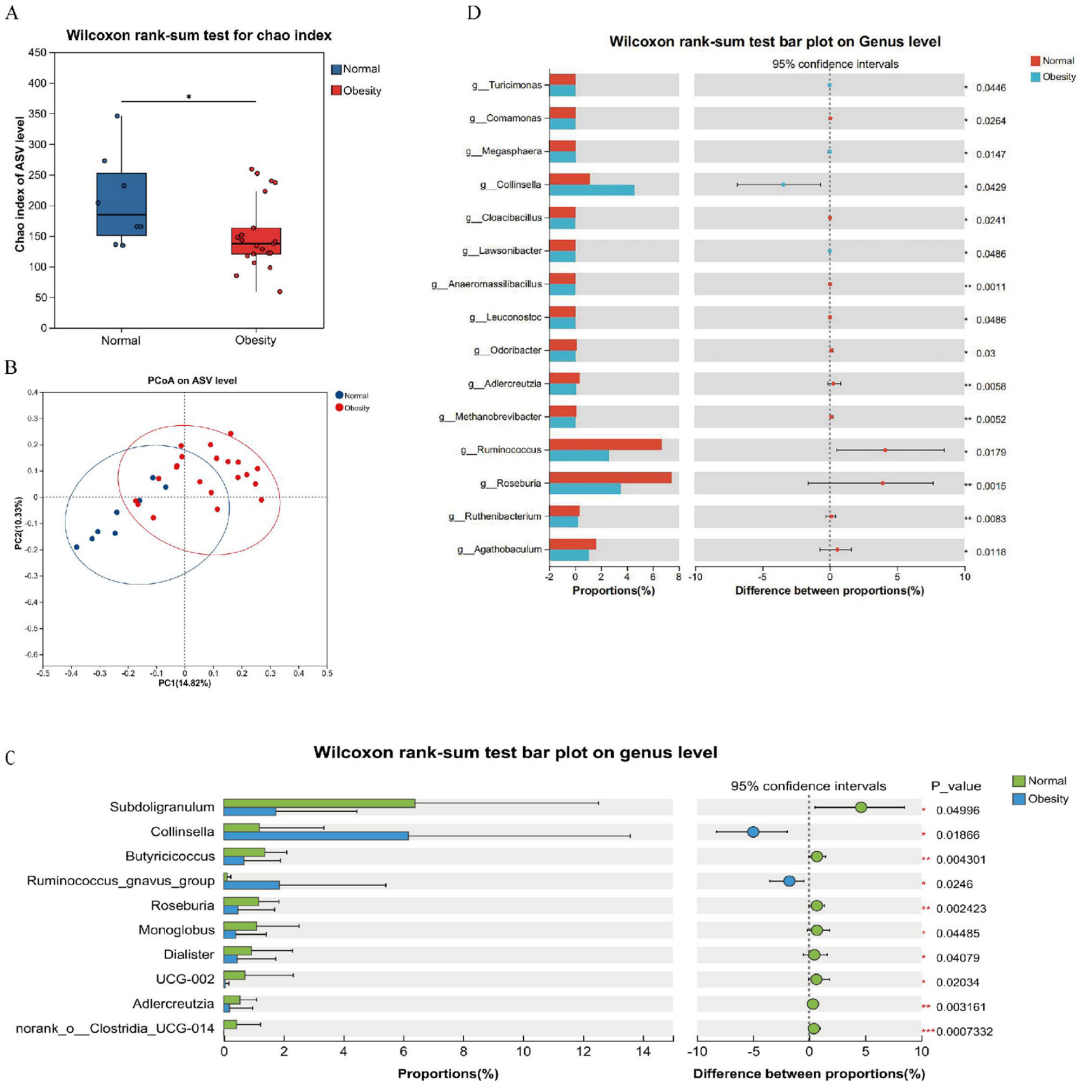
Structural analysis of the intestinal flora. **(A)** Alpha diversity chao index. **(B)** PCoA based on unweighted Unifrac distance used to show beta diversity between groups. **(C)** Analysis of the top 10 significantly different species from 16s rRNA abundance. **(D)** Analysis of significantly different species in the top 15 of abundance from macrogenomes. *p < 0.05, **p < 0.01, ***p < 0.001.

The alterations in gut microbial composition were initially examined between normal controls and obese patients. It was observed that microbial abundance was significantly diminished in the obese cohort (Figure 2A). However, the Shannon index showed no significant difference (Supplementary Figure S1). Furthermore, Principal Coordinates Analysis (PCoA) at the Amplicon Sequence Variant (ASV) level indicated a divergence in the microbiota structure of obese individuals compared to normal controls (Figure 2B). Differential analyses of bacterial flora at the genus level, utilizing 16S rRNA and metagenomic sequencing, identified several microorganisms that exhibited significant differences between healthy and obese groups. These differential bacteria, particularly *Subdoligranulum, Klebsiella, g__Roseburia*, and *g__Ruminococcus*, may serve as potential biomarkers for distinguishing obesity status (Figures 2C, D). Metagenomic analysis further examined species differences among the top 10 species by abundance at the genus level (Supplementary Figure S2). It was found that *s__Faecalibacterium_prausnitzii, s__Ruminococcus_bromii, s__Roseburia_faecis, s__Alistipes_putredinis* were predominantly distributed in the normal and grade 1 obesity groups. Notably, *s__Alistipes_putredinis* was not observed in other groups. *s__Bacteroides_stercoris* was almost exclusively enriched in grade 1 obesity and may represent a potential biomarker. Ruminococcus gnavus exhibited higher abundance in the grade 2 obesity group. The species *s__Collinsella_aerofaciens* showed elevated abundance in overweight and grade 3 obesity groups.

### 3.3 Biomarker analysis of health and obesity

**Figure 3:**
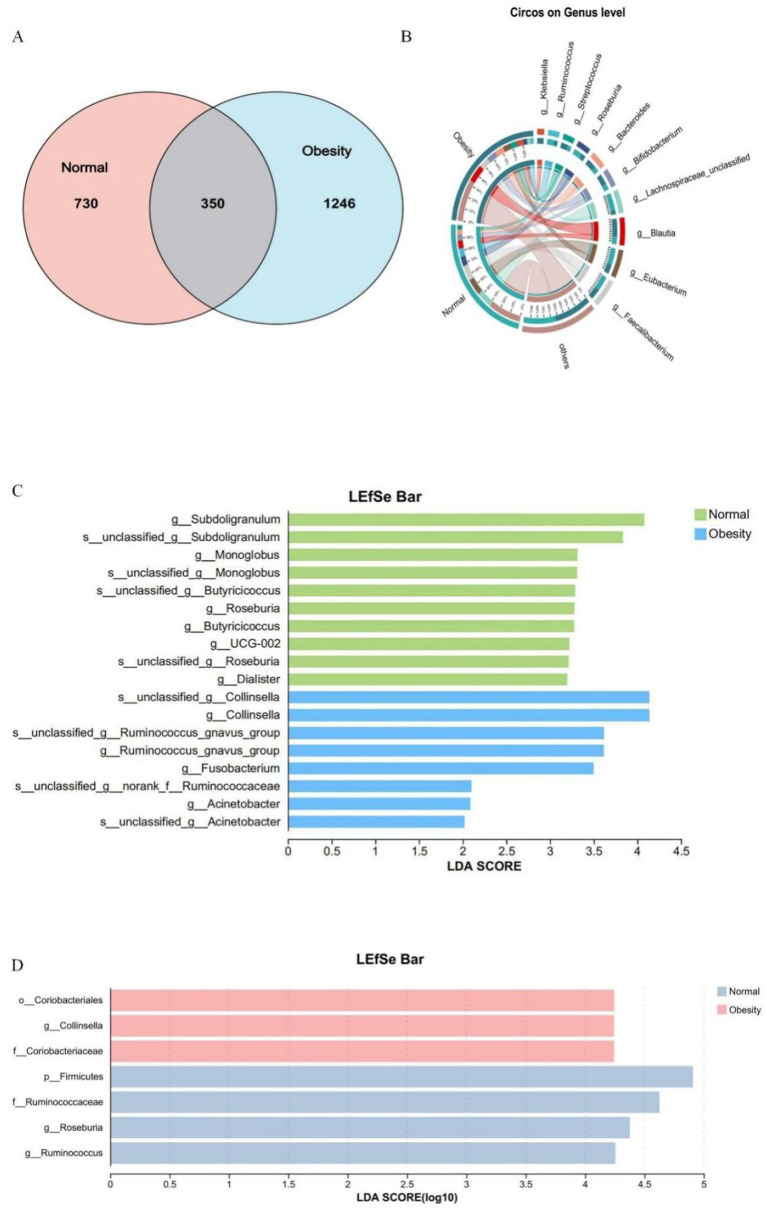
Normal weight and obesity biomarker analysis. **(A)** Venn diagram of shared or unique ASVs between healthy controls and obese patients. **(B)** Heatmap of species composition at the genus level in healthy controls and obese patients. **(C)** Linear discriminant analysis of 16SrRNA LEfSe, LDA score > 2.0. **(D)** Linear discriminant analysis of LEfSe in the macrogenome, LDA score > 4.0.

To investigate the microorganisms potentially influencing obesity progression and to identify biomarkers for distinguishing obesity, a species Venn analysis at the Amplicon Sequence Variant (ASV) level was conducted. This analysis revealed that 730 ASVs were unique to healthy controls, 1,246 ASVs were unique to obese individuals, and 350 ASVs were common to both groups (Figure 3A). Further analysis of species composition at the ASV level, along with Linear Discriminant Analysis (LDA) Effect Size (LEfSe) for the top ten species based on genus-level richness, demonstrated an increase in the genera *g__Blautia* (70%), *g__Streptococcus* (67%), *g__Bacteroides* (67%), and *g__Bifidobacterium* (64%) in obese subjects. Conversely, there was a decrease in the genera *g__Ruminococcus (28%), g__Roseburia* (32%), *g__Lachnospiraceae_unclassified* (19%), *g__Eubacterium* (34%), and *g__Faecalibacterium* (41%). Notably, the genus *g__Klebsiella* was specific to obesity (Figure 3B). Consistent findings from 16S rRNA and metagenomic LEfSe analyses identified *g__Collinsella* as an obesity marker, whereas *g__Roseburia* and *g__Ruminococcus* were identified as markers of health (Figures 3C, D).

### 3.4 Gut microbial characteristics of patients with different levels of obesity

**Figure 4:**
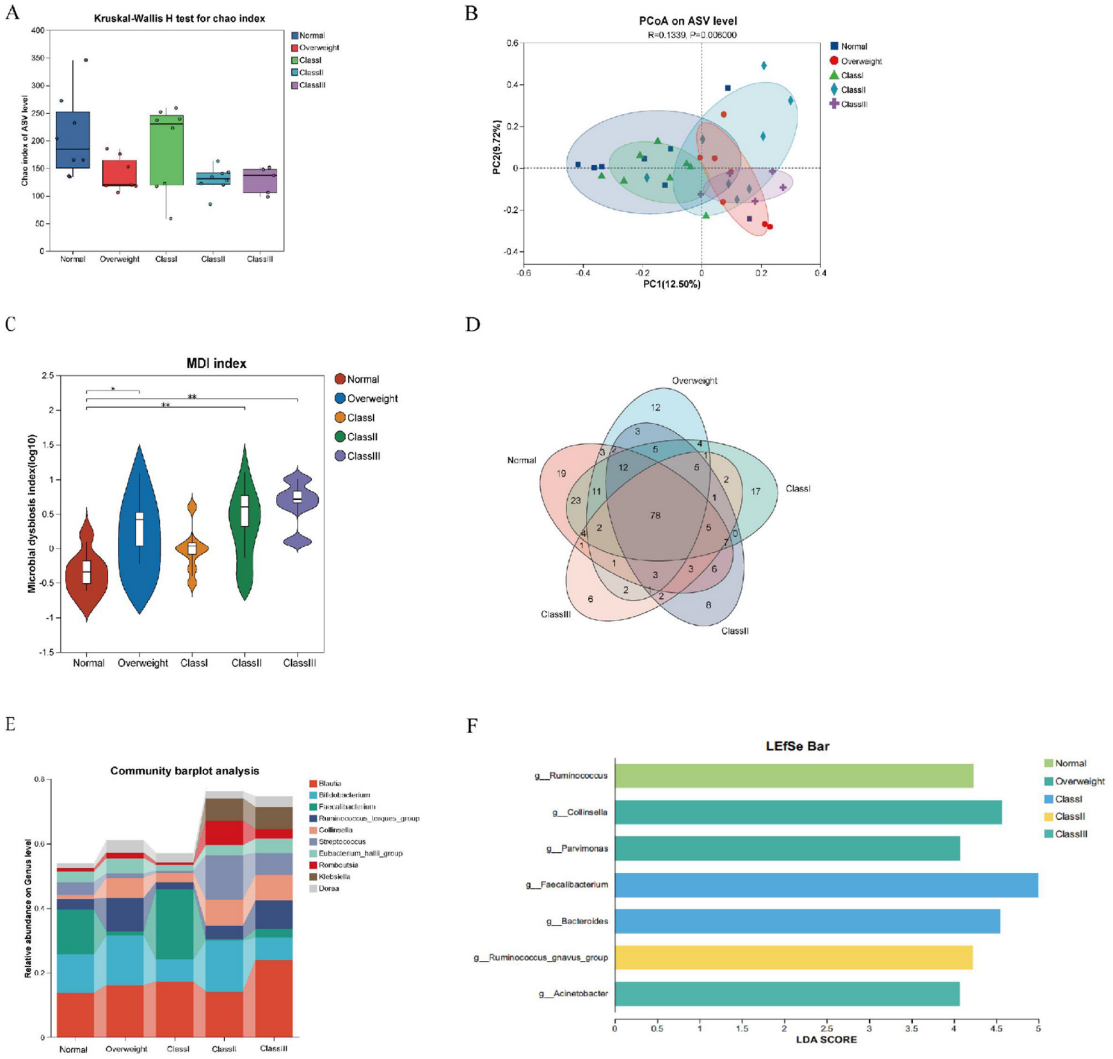
Characterization of gut flora in healthy, overweight and class I, II and III obese patients. **(A)** Alpha diversity chao index for the five groups. **(B)** PCoA analysis at the ASV level, demonstrating the degree of similarity and difference in the microbial communities of different groups. **(C)** Venn diagram at the genus level, reflecting species shared or endemic. Different colors represent different subgroups, overlapping parts indicate species common to multiple subgroups, parts without overlap indicate species endemic to the subgroup, and numbers indicate the corresponding number of species. **(D)** Genus-level colony structure disorder index (MDI). **(E)** Genus-level species composition analysis. **(F)** Genus-level Lefse species difference discriminant analysis with LDA score > 4.0. *p < 0.05; **p < 0.01.

To comprehensively examine the intestinal microbiota composition across varying degrees of obesity, subjects were stratified into categories of overweight, and Obesity Classes I, II, and III. Analysis of alpha diversity using the Chao index, along with Principal Coordinates Analysis (PCoA) at the species abundance value (SAV) level, demonstrated significant alterations in microbiota composition among the different groups (Figures 4A, B). Furthermore, a Venn diagram at the genus level indicated a decline in the number of endemic species as obesity severity increased (Figure 4C). The microbial dysbiosis index (MDI) progressively rose, indicating a worsening of microbial community structure disorder (Figure 4D). Notably, the Class I obesity group did not exhibit significant differences. To elucidate species-specific alterations, the top ten species in terms of genus-level abundance were analyzed in relation to obesity levels. The analysis revealed that *Collinsella, Streptococcus, Blautia, Romboutsia*, and *Klebsiella* exhibited an increasing trend with obesity, whereas *Faecalibacterium* demonstrated a decreasing trend. Notably, the microbial structure of the Class I obesity group appeared to revert toward a normal configuration (Figure 4E and Supplementary Figure S3), indicating that the microbial community structure may undergo compensatory regulation during the progression of obesity. Further investigation into potential biomarkers of obesity severity among the top ten species revealed that *Ruminococcus* served as a marker for the normal group; *Collinsella* and *Parvimonas* were indicative of the overweight group; *Faecalibacterium* and *Bacteroides* were associated with the Class I obesity group; *Ruminococcus_gnavus_group* was identified as a marker for Class II obesity; and *Acinetobacter* was linked to Class III obesity (Figure 4F).

### 3.5 Functional predictive analysis

**Figure 5:**
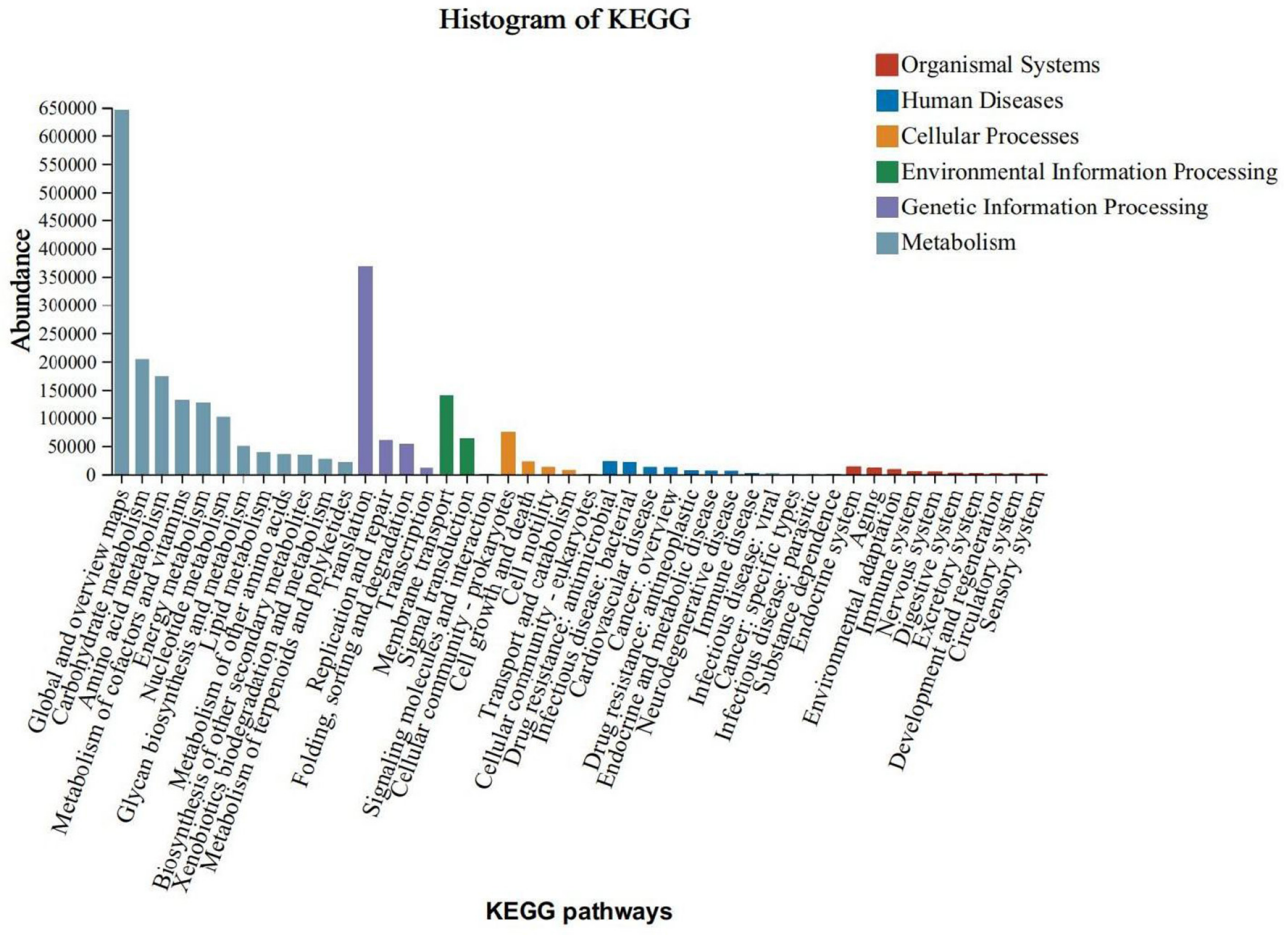
KEGG Functional Annotation Analysis.

**Figure 6:**
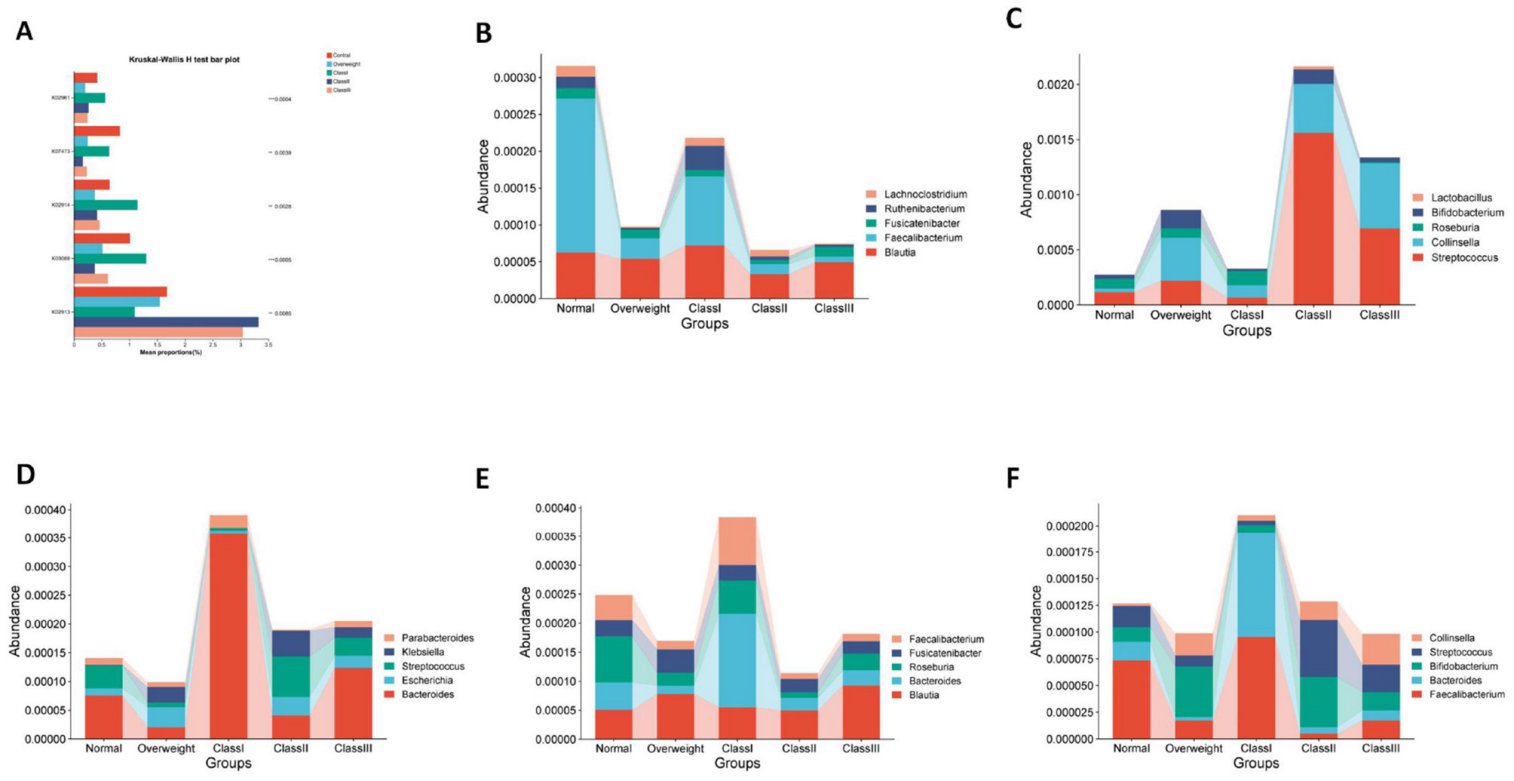
Functional prediction analysis. **(A)** Differences in the top five species/functional abundances KO. **(B)** Large subunit ribosomal protein L33, **(C)** RNA polymerase sigma-70 factor, ECF subfamily. **(D)** Large subunit ribosomal protein L34. **(E)** DNA-damage-inducible protein J. **(F)** Small subunit ribosomal protein S17.

The KEGG functional annotation predominantly enriched the Global and Overview Maps, Carbohydrate Metabolism, and Amino Acid Metabolism functions within the Metabolic Processes pathway. It also enriched the Translation function within the Genetic Information Processing pathway, the Membrane Transport function within the Environmental Information Processing pathway, and the Cellular Community - Prokaryotes function within the Cellular Processes pathway. Notably, the enrichment was more pronounced in the Metabolic Processes pathway (Figure 5).To predict functional differences attributable to colony contributions across varying levels of obesity, a functional difference analysis was conducted on the top five species abundances. Additionally, an analysis of species contributions to these top five abundances was performed at the KEGG Orthology (KO) functional level. The findings indicated significant variations in K02913 (L33 ribosomal protein), K03088 (σ70 RNA polymerase), K02914 (L34 ribosomal protein), K07473 (DNA damage repair protein J), and K02961 (S17 ribosomal protein) across different stages of obesity (Figure 6A).The L33 protein (K02913) analysis revealed that the normal and grade I obesity groups were predominantly influenced by *Faecalibacterium*, whereas *Blautia* was the principal contributor in the overweight and moderately severe obesity groups (grade II/III) (Figure 6B). In the case of the σ70 RNA polymerase (K03088), *Collinsella* and *Streptococcus* were the primary contributors in grade II/III obesity, with *Collinsella* also being predominant in the overweight group (Figure 6C). For the L34 protein (K02914), *Bacteroides* was the major contributor in the normal, class I, and class III obesity groups, while class II obesity was primarily influenced by *Streptococcus* (Figure 6D). The DNA damage repair protein J (K07473) analysis indicated that *Roseburia* was dominant in the normal group, *Blautia* in the overweight and moderate obesity groups, and Bacteroides in the primary obesity group (Figure 6E). Lastly, the S17 ribosomal proteins (K02961) analysis showed that *Faecalibacterium* was the main contributor in the normal and primary obesity groups, Bifidobacterium was predominant in the overweight and secondary obesity groups, and *Collinsella* was most prevalent in the tertiary obesity group (Figure 6F).

In conclusion, the study identifies *Blautia* as frequently exhibiting dominant functions in individuals with overweight and moderate-to-severe obesity, such as the L33 protein and DNA repair protein J, suggesting its adaptation to obesity-related metabolic stresses. Conversely, *Faecalibacterium* is predominantly found in individuals with normal weight and mild obesity, associated with proteins like L33 and S17, and appears to play a role in maintaining metabolic homeostasis; its reduction may correlate with the progression of obesity. Additionally, *Collinsella* and *Streptococcus* are significantly implicated in RNA polymerase and ribosomal functions in cases of severe obesity, potentially linking them to metabolic disorders or inflammation. *Roseburia*, associated with DNA repair in the normal weight group, may offer protective benefits, with its reduced abundance potentially linked to obesity progression. Overall, the grade of obesity is intricately connected to the functional contributions of intestinal flora. Genera such as *Blautia, Collinsella*, and *Streptococcus* may adapt to host metabolic changes by modulating ribosome synthesis and stress responses, positioning them as potential microbial markers for obesity classification or as targets for therapeutic intervention.

### 3.6 Correlation between intestinal flora and biochemical indicators in patients with different degrees of obesity

**Figure 7:**
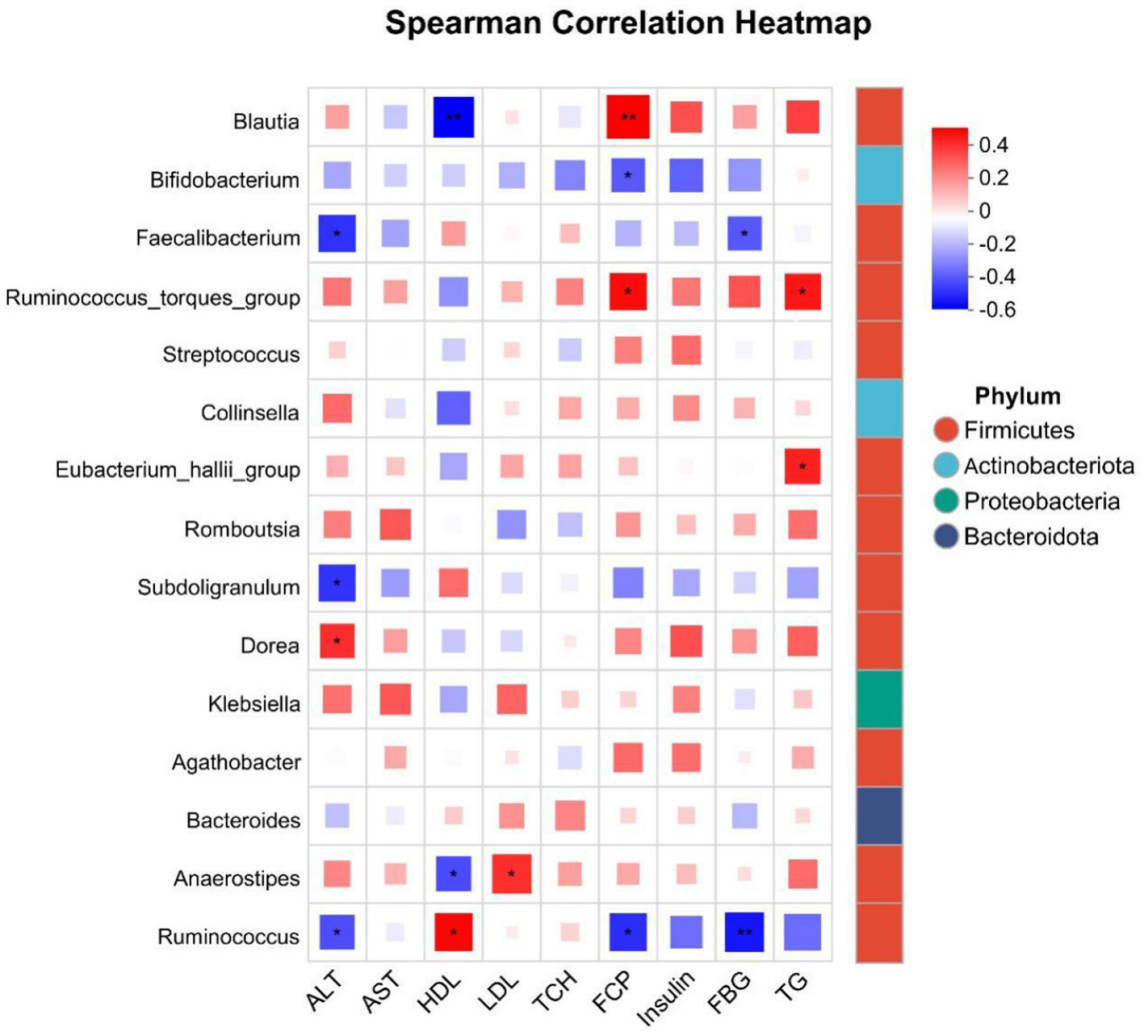
Correlation analysis of the top 20 species abundance with biochemical indicators. *P < 0.05, **P < 0.01.

Given the observed variations and trends in biochemical indicators among overweight and obese patients, we aimed to establish correlations between these indicators and intestinal flora. To achieve this, we analyzed the top 15 species based on species abundance for their correlation with biochemical indicators. Our findings revealed that these indicators were influenced by one or multiple intestinal flora, with the primary focus on identifying flora that could serve as potential biomarkers. Specifically, ALT, FCP, and FBG showed significant negative correlations with *Ruminococcus*, a biomarker of health, and significant positive correlations with HDL. Additionally, ALT and FBG were significantly negatively correlated with *Faecalibacterium*, identified as a biomarker for Class I obesity. Although *Collinsella* was not significantly correlated with these clinical factors as a marker of overweight, AST and HDL exhibited a tendency for negative correlation with *Collinsella*, while other factors showed a tendency for positive correlation. Furthermore, HDL demonstrated a highly significant negative correlation with *Blautia*, and FCP showed a highly significant positive correlation with *Blautia* (Figure 7).

## 4 Discussions

As the severity of obesity progresses from overweight to tertiary obesity, there is a notable decrease in the diversity of intestinal microbiota, accompanied by a gradual increase in the dysbiosis index (MDI). Interestingly, the alterations in microbiota composition observed in individuals with tertiary obesity closely resemble those found in healthy individuals, suggesting the potential presence of compensatory regulatory mechanisms during the early stages of obesity. Previous studies have demonstrated a significant reduction in intestinal microbiota diversity with increasing obesity, which may be linked to obesity-related metabolic disorders (Florêncio et al., 2025; Xie et al., 2025). It is hypothesized that in the initial phases of obesity, compensatory regulation of the intestinal microbiota may occur as an adaptive response to changes in the internal environment (Maqoud et al., 2024). Specifically, the gut microbiota of obese individuals is typically characterized by a higher proportion of Firmicutes and a lower proportion of Bacteroidetes, a shift that is believed to be associated with enhanced energy uptake and storage (Enache et al., 2024; Mamun et al., 2025). Furthermore, the presence of certain pro-inflammatory bacteria within the gut microbiota of obese individuals may exacerbate metabolic complications associated with obesity by compromising intestinal barrier integrity and promoting systemic inflammation (Maqoud et al., 2024; Custers et al., 2024).

During the initial stages of obesity, alterations in the gut microbiota may be subtle or even resemble those observed in healthy individuals. This phenomenon could be attributed to the body's ability to maintain gut microbiota equilibrium through compensatory mechanisms in the early phases of obesity (Deng et al., 2025). However, as obesity progresses, these compensatory mechanisms may become insufficient, resulting in reduced microbial diversity and an elevated dysbiosis index (MDI) (Xie et al., 2025). In summary, alterations in gut microbiota are pivotal in the pathogenesis of obesity, and elucidating these changes is crucial for the development of novel therapeutic strategies for obesity management.

A decline in beneficial bacteria, such as *Faecalibacterium* and *Roseburia*, alongside an increase in pathogenic bacteria, including *Blautia* and *Collinsella*, is correlated with the progression of obesity. *Faecalibacterium* and *Roseburia* are recognized for their production of short-chain fatty acids (SCFAs), particularly butyrate, which is crucial for maintaining gut barrier integrity and exerting anti-inflammatory effects. A reduction in these bacteria may compromise intestinal barrier function, potentially leading to chronic inflammation, a key pathophysiological mechanism underlying obesity and metabolic syndrome (Duan et al., 2021). Furthermore, the proliferation of pathogenic bacteria like *Blautia* and *Collinsella* is linked to metabolic abnormalities associated with obesity. The expansion of these microbial populations may disrupt intestinal microecological balance, thereby influencing host energy and lipid metabolism, and promoting adiposity and weight gain (Companys et al., 2021). Additionally, increased levels of *Collinsella* have been positively correlated with body fat and low-density lipoprotein (LDL) levels in obese individuals, underscoring its potential contribution to the pathogenesis of obesity (Zeng et al., 2019). In conclusion, alterations in the composition of the intestinal microbiota, particularly the reduction of beneficial bacteria and the proliferation of pathogenic bacteria, constitute a significant factor in the development and progression of obesity.

The functions of the microbiota in obese individuals are skewed toward ribosome synthesis and stress response pathways (e.g., K02913, K03088), indicating potential mechanisms of metabolic adaptation. This phenomenon suggests a significant role in metabolic adaptation, as the gut microbiota in an obese state may adjust to the host's metabolic demands by upregulating the ribosome synthesis pathway. Such an adaptation could be associated with the increased protein synthesis needs of cells in response to external environmental changes. In the context of stress response, bacteria typically modulate ribosomal functions to cope with environmental stresses. For instance, under heat stress conditions, bacterial ribosomes may selectively bind specific mRNAs, thereby regulating protein synthesis (Fisunov et al., 2017). This selective binding enables bacteria to swiftly modify their metabolic activities, ensuring survival and functionality under adverse conditions. Furthermore, the gut microbiota of obese individuals may influence the host's metabolic status by modulating ribosome-related pathways. Increased ribosome synthesis has been implicated in the development of obesity-associated metabolic disorders, potentially due to disruptions in protein synthesis that can adversely affect normal cellular functions and metabolic processes (Schöttl et al., 2020). Consequently, a comprehensive investigation into the role of gut microbiota in ribosome synthesis and stress response among obese individuals could offer novel insights into the mechanisms underlying obesity-related metabolic diseases.

Specific bacterial genera, such as *g__Faecalibacterium* and *g__Bacteroides* (class I), *g__Ruminococcus_gnavus_group* (class II), and *Acinetobacter* (class III), have been identified as markers indicative of obesity severity. Research indicates a strong association between these genera and metabolic abnormalities linked to obesity. For instance, there is a notable negative correlation between *Faecalibacterium, prausnitzii* and obesity-related metabolic markers, suggesting that this bacterium may play a crucial role in maintaining gut health and metabolic homeostasis (Brahe et al., 2015). Certain species within the genus *Bacteroides* exhibit varying abundance in obese individuals, potentially correlating with the host's metabolic status (Zeng et al., 2019).

Significant alterations in the composition and functionality of the gut microbiota can manifest at various stages of obesity. Research indicates that the gut microbiota of obese individuals is characterized by an increased proportion of Firmicutes and a decreased proportion of Bacteroidetes, a shift linked to enhanced energy absorption and fat storage (Krznarić et al., 2012). Furthermore, specific alterations in the genus Acinetobacter have been observed in individuals with grade III obesity, potentially correlating with more severe metabolic disturbances (Kong et al., 2013). The progression of obesity is also associated with modifications in other microbial populations. Studies have demonstrated that obese individuals generally exhibit reduced gut microbial diversity, which may contribute to impaired metabolic function (De la Cuesta-Zuluaga et al., 2018). Additionally, alterations in certain microbial taxa may be linked to obesity-related inflammatory responses, further exacerbating metabolic disturbances (Moreno-Indias et al., 2016). Moreover, the genus *Blautia* has been positively associated with insulin resistance, while *Faecalibacterium* has been positively correlated with high-density lipoprotein (HDL) levels, suggesting their potential as biomarkers for obesity diagnosis (Naderpoor et al., 2019). Firstly, concerning the association between *Blautia* and insulin resistance, research demonstrates a positive correlation between the abundance of *Blautia* and insulin resistance. One study found a significant association between *Blautia* abundance and insulin resistance in obese children, which was also correlated with increased levels of inflammatory markers, including TNF-α and IL-6 (Benítez-Páez et al., 2020; Zhu et al., 2020). Additionally, another study suggested that a reduction in *Blautia* abundance might be linked to obesity-related metabolic inflammation, thereby reinforcing the connection between *Blautia* and insulin resistance (Benítez-Páez et al., 2020). Secondly, the positive correlation between *Faecalibacterium* and high-density lipoprotein (HDL) levels has been corroborated by multiple studies. *Faecalibacterium prausnitzii*, a butyrate-producing bacterium, shows a positive correlation between its abundance and elevated HDL levels (Gruneck et al., 2020; Kashtanova et al., 2021). Research indicates that increased *Faecalibacterium* abundance is associated with improved metabolic health, potentially due to its production of short-chain fatty acids, such as butyrate, in the gut, which may enhance lipid metabolism (Gruneck et al., 2020). Finally, the positive correlation between *Collinsella* species and LDL cholesterol levels has been substantiated by multiple studies. One study reported a higher abundance of *Collinsella* aerofaciens in overweight and obese individuals, which was positively correlated with LDL levels (Companys et al., 2021). Furthermore, another study proposed that an increase in *Collinsella* may be linked to metabolic disorders, possibly through its influence on cholesterol metabolic pathways (Reimer et al., 2021). These genera exhibit distinct alterations at various stages of obesity, which may be intricately linked to the severity of obesity and its associated metabolic abnormalities (Chakaroun et al., 2021; Hu et al., 2024; Huang et al., 2023; Jie et al., 2021; Mohammadzadeh et al., 2024; Schwenger et al., 2025). In conclusion, the significance of the gut microbiota in metabolic health is garnering heightened scholarly interest. The correlation between specific bacterial taxa, including *Blautia, Faecalibacterium*, and *Collinsella*, and various metabolic markers provides novel insights into the involvement of gut microbiota in metabolic disorders. These findings contribute to a deeper understanding of the intricate relationship between gut microbiota and metabolic health and identify potential targets for future therapeutic interventions.

In summary, the present study conducted a systematic analysis of the gut microbiota characteristics among individuals with varying obesity levels (healthy, overweight, Grade I, Grade II, and Grade III obesity) in Southwest China, utilizing 16S rRNA gene sequencing and metagenomic sequencing technologies. This analysis has identified potential biomarkers for the microbiological diagnosis of obesity and has suggested novel approaches for the treatment of obesity and related metabolic disorders through the modulation of the intestinal microbiota. Nonetheless, this study is subject to several limitations. the most significant of which is the relatively small sample size and no discussion of gender differences was conducted. This limitation primarily arises from challenges in recruiting severely obese patients and healthy controls who fulfill stringent inclusion criteria, as well as the high costs associated with metagenomic sequencing analysis for each sample. The restricted sample size may compromise statistical power, heighten the risk of Type II errors, and potentially broaden the confidence intervals for effect estimates. To enhance the robustness of our findings, we conducted a review of the relevant literature and performed comparative studies, which revealed a reduction in microbial diversity within the obese population. Notably, we observed specific alterations in certain genera, including an increase in *Blautia* and *Collinsella* among obese individuals, alongside a decrease in *Faecalibacterium* and *Roseburia* (Supplementary Tables 1, 2). This study distinguishes itself by further refining specific biomarkers associated with varying levels of obesity—namely, *Ruminococcus* for normal weight, *Collinsella* for overweight, *Faecalibacterium* for Class I obesity, *Ruminococcus_gnavus_group* for Class II obesity, and Acinetobacter for Class III obesity. These findings elucidate a close relationship between the identified biomarkers and host metabolic functions, such as ribosome synthesis and RNA polymerase activity, offering a more in-depth analysis than previous research. Consequently, this study should be regarded as preliminary and exploratory, as it provides valuable targets and directions for future mechanistic investigations. However, the generalizability of these findings requires validation through studies involving larger and more representative population cohorts.

## 5 Conclusion

Populations exhibiting varying obesity profiles demonstrate distinct microbiota compositions, with flora adapted to obesity, and an escalation in dysregulation of these microbial communities is observed with increasing levels of obesity. There exists a correlation between alterations in microbial functionality and host metabolic processes, including RNA polymerase activity, DNA damage repair, and metabolic pathways such as ribosome synthesis, insulin resistance, glucose metabolism, and lipoprotein regulation. Targeted interventions aimed at modulating microbiota that are significantly elevated in obesity (e.g., *Collinsella*) and augmenting those significantly reduced in non-obese individuals (e.g., *Faecalibacterium*) may represent an effective strategy for mitigating obesity.

## Data Availability

The data presented in this study have been deposited in the figshare repository: 10.6084/m9.figshare.30529985.
