# Supplementary material for: Characterization of the gut microbiota in people with different levels of obesity
Source: Front Microbiol. 2025 Dec 3;16:1679119. doi: 10.3389/fmicb.2025.1679119 (PMC12709931; doi:10.3389/fmicb.2025.1679119)
Supplement: Supplementary file 1 [file Supplementary_file_1.docx]

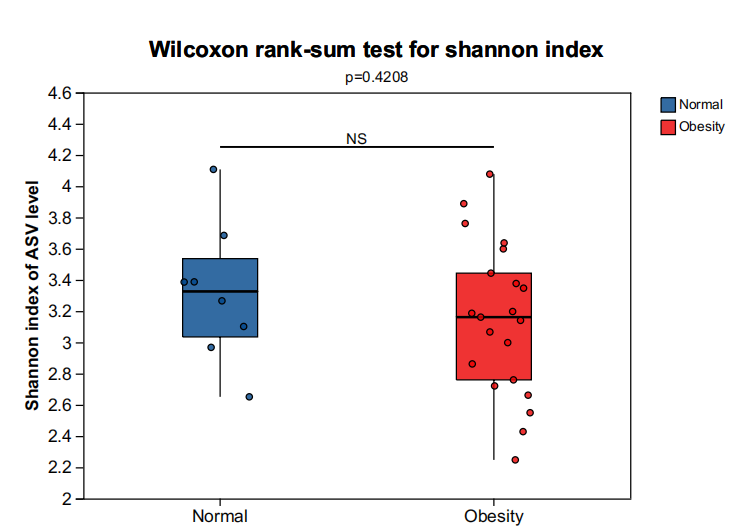


Supplementary Figure S1 based on α analysis of the Shannon index.


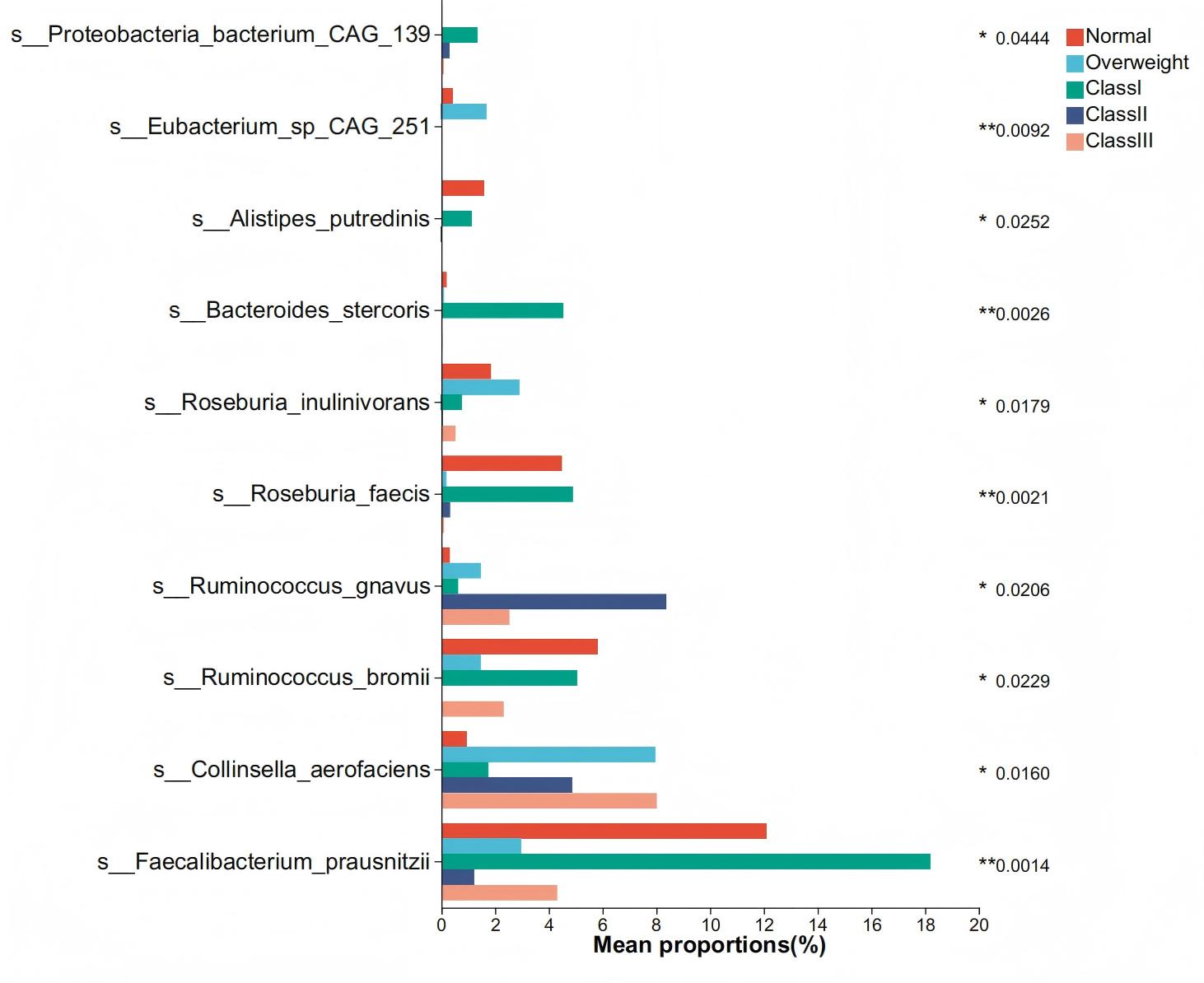


Supplementary Figure S2 Analysis of Species Differences at the Species Level


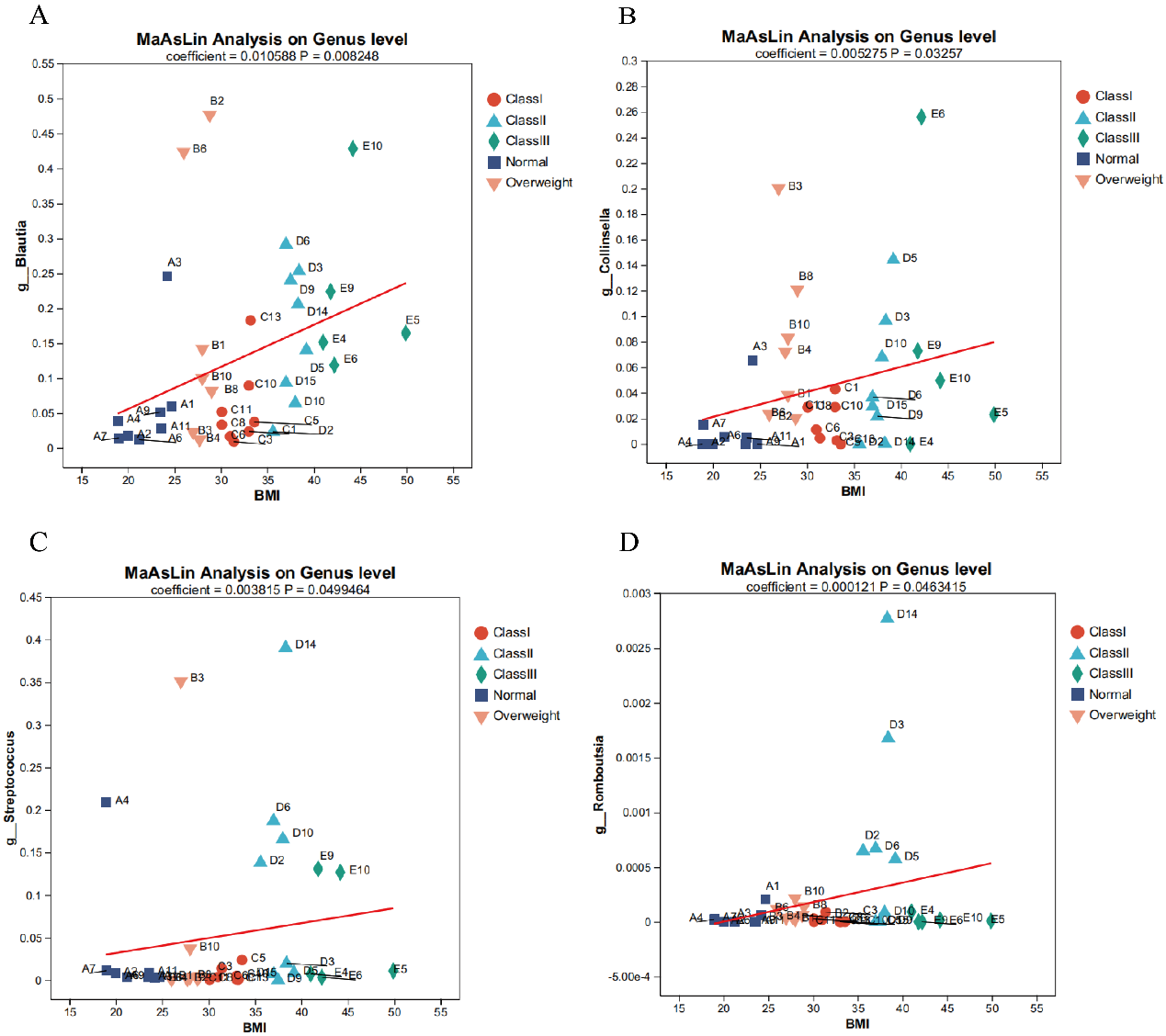


Supplementary Figure S3 Correlation analysis between BMI and species Blautia (A), Collinsella (B), Streptococcus (C), and Romboutsia (D). X-axis represents BMI, Y-axis represents relative abundance of species; Coefficient: Magnitude of correlation coefficient between environmental variables and species (greater than 0 indicates positive correlation, less than 0 indicates negative correlation, equal to 0 indicates no correlation); p-value measures the reliability of the test, with p < 0.05 indicating a significant correlation between environmental variables and species.

**Supplementary Tables 1 Microbial Biomarkers Identified in Different Obesity Classifications**

| Group | Biomarker | Taxonomic Level |
| --- | --- | --- |
| Normal | Eubacterium coprostanoligenes group, Tannerellaceae | Family |
|  | *Eubacterium coprostanoligenes group Lachnospiraceae NK4A136 group, Parabacteroides*  *Akkermansia* | Genus |
|  | *Prevotella cop，Bacteroides ovatus* | Species |
| Overweight | Gammaproteobacteria | Class |
|  | Enterobacterales | Order |
| Class I obesity | Erysipelatoclostridiaceae | Family |
| Class II obesity | Bacilli | Class |
|  | Lactobacillales | Order |
| Class III obesity | Negativicutes | Class |

**Note:** These biomarkers reveal that as the severity of obesity increases, the gut microbiota transitions from a predominantly beneficial population that produces short-chain fatty acids (healthy group) to a population characterized by associations with inflammation, opportunistic pathogens, and energy metabolism disorders.

**Method of identification:** LEfSe, Linear discriminant analysis Effect Size; RF, Random Forest. **Source:** Hu et al. Gut microbiota Signature of Obese Adults Across Different Classifications. Diabetes Metab Syndr Obes. 2022;15:3933-3947.

**Supplementary Tables 2 Microbial and Functional Biomarkers Associated with Obesity Identified from Metagenomic Analysis**

| Category | Biomarker Description | Association with Obesity |
| --- | --- | --- |
| Phylum-Level Taxonomy | Actinobacteria | Enriched |
|  | Bacteroidetes | Depleted |
| Functional Genes | Phosphotransferase System (PTS) genes | Enriched |
|  | Carbohydrate Metabolism pathways | Enriched (in Bacteroidetes bins from lean subjects) |
|  | Transport Systems | Enriched (in Firmicutes bins) |
| Gene Ontology | 383 genes (vs. custom 44-genome database) | Differentially Abundant (q<0.05) |
|  | 273 genes | Enriched in Obese Microbiome |
|  | 110 genes | Depleted in Obese Microbiome |
| Taxonomic Origin of Enriched Genes | Actinobacteria | Source of 75% of obesity-enriched genes |
|  | Firmicutes | Source of 25% of obesity-enriched genes |
| Taxonomic Origin of Depleted Genes | Bacteroidetes | Source of 42% of lean-enriched (obese-depleted) genes |
| Functional Annotation | Genes involved in carbohydrate, lipid, and amino acid metabolism | Differentially Abundant |

**Notes:**

- **Enriched/Depleted:** Terms refer to the relative abundance in the gut microbiome of obese individuals compared to lean individuals.
- **Source:** Turnbaugh et al. A core gut microbiome in obese and lean twins. Nature. 2009;457(7228):480-484.
- The findings are based on the analysis of fecal microbiomes from monozygotic and dizygotic twin pairs concordant for leanness or obesity, and their mothers.

**Key Summary from the Paper:** This seminal study found that obesity in humans is associated with:

1. **Phylum-level changes:** An increase in Actinobacteria and a decrease in Bacteroidetes.
2. **Reduced bacterial diversity** in the gut.
3. **Altered functional capacity:** The obese microbiome is enriched for genes involved in processing dietary carbohydrates (e.g., phosphotransferase systems), likely increasing the energy harvest potential from the diet.
4. **A functional core microbiome:** While a core set of microbial species was not found across all individuals, a core set of microbial genes and metabolic functions was identified. Deviations from this functional core are associated with the obese state.
